# Unstructured Electronic Health Records of Dysphagic Patients Analyzed by Large Language Models

**DOI:** 10.1109/JTEHM.2025.3571255

**Published:** 2025-05-19

**Authors:** Luisa Neubig, Deirdre Larsen, Melda Kunduk, Andreas M. Kist

**Affiliations:** Department of Artificial Intelligence in Biomedical EngineeringFriedrich-Alexander-Universität Erlangen-Nürnberg Erlangen 91054 Germany; Department of Communication Sciences and DisordersEast Carolina University 3627 Greenville NC 27858 USA; Department of Communication Sciences and DisordersLouisiana State University 5779 Baton Rouge LA 70802 USA

**Keywords:** Dysphagia, EHR, clustering analysis, natural language processing

## Abstract

Objective: Dysphagia is a common and complex disorder that complicates both diagnoses and treatment. Consequently, the associated electronic health records (EHR) are often unstructured and complex, posing challenges for systematic data analysis.Methods and procedures: In this study, we employ natural language processing (NLP) techniques and large language models (LLMs) to automatically analyze clinical narratives and extract diagnostic information from a diverse set of EHRs. Our dataset includes medical records from 486 patients, representing a group with diverse dysphagic conditions. We analyze diagnoses provided in unstructured free text that do not follow a standardized structure. We utilize clustering algorithms on the extracted diagnostic features to identify distinct groups of patients who share similar pathophysiological swallowing dysfunctions.Results: We found that basic NLP techniques often provide limited insights due to the high variability of the data. In contrast, LLMs help to bridge the gap in understanding the nuanced medical information about dysphagia and related conditions. Although applying these advanced LLM models is not straightforward, our results demonstrate that leveraging closed-source models can effectively cluster different categories of dysphagia.Conclusion: Our study provides therefore evidence that LLMs are highly promising in future dysphagia research.Clinical impact: Dysphagia is a symptom associated with various diseases, though its underlying relationships remain unclear. This study demonstrates how analyzing large volumes of electronic health records can help clarify the causes of dysphagia and identify contributing factors. By applying natural language processing, we aim to enhance both understanding and treatment, supporting clinical staff in improving individualized care by identifying relevant patient cohorts. Clinical and Translational Impact Statement: This study uses LLMs to efficiently preprocess unstructured EHRs, improving dysphagia diagnosis and patient clustering. It aligns with Clinical Research, enhancing diagnostic speed and enabling personalized treatment.

## I. Introduction

Accurate and efficient communication of medical diagnoses is essential for healthcare. However, one of the challenges in the medical literature and patient-associated reports lies in the diverse and sometimes inconsistent terminology and abbreviations used to describe similar pathological conditions. This problem is especially evident in the area of dysphagia, where various terms may describe the same or similar clinical situations. Dysphagia refers to any difficulty in swallowing, involving the transport of food or liquids from the mouth to the stomach. These difficulties may impact sensory or motor functions during the swallowing process. Therefore, dysphagia is more a symptom, associated with a range of underlying diseases, than a standalone diagnosis [1]. Electronic health records (EHR) are essential for the management and storage of medical records. These digital records contain comprehensive patient information ranging from diagnostic outcomes to therapeutic interventions [2]. The use of EHRs has significantly changed how healthcare providers access and use patient data, enhancing the efficiency of medical decision-making. The EHR contains a written diagnosis in the note and an associated diagnosis selected and documented in the larger medical record. This usually consists of ICD-10 codes, which are standard formulations used for billing and contribute to a summarized list of historical diagnoses [3]. In this work, we analyze only descriptive diagnoses based on unstructured free text. However, the large volume of data in EHRs brings both benefits and challenges. While the comprehensive collection of patient health information is valuable, it also places considerable demands on healthcare systems, requiring sophisticated methods to manage and analyze the data effectively [4]. To address the challenges associated with managing large datasets in healthcare, the use of machine learning techniques has become increasingly common. Machine learning algorithms enable the analysis of extensive data sets, thereby improving the accuracy and efficiency of medical diagnoses [5]. These technologies enable the extraction of essential insights from the complex and voluminous data contained in EHRs, enhancing patient care through timely and customized medical interventions [6]. Furthermore, the analysis of unstructured free text in EHRs poses significant difficulties due to the variability in terminology and the presence of ambiguities in language [7]. This study introduces a methodological framework that employs natural language processing (NLP), and especially Large Language Models (LLMs) to address these challenges. By integrating LLMs, our proposed framework aims to standardize the interpretation of diverse medical terms and abbreviations, enhancing the reliability of diagnostic data used in subsequent analytical tasks. The cognitive capabilities of LLMs are leveraged to improve understanding and mitigate the issues posed by unstructured free text analysis in medical documents. Specifically, we aimed to utilize LLMs to cluster dysphagic pathological cases into distinct groups, revealing underlying patterns and commonalities that may not be immediately apparent when relying solely on traditional diagnostic terminology.

## II. Related Works

Data mining in EHRs is a common strategy to understand the course of diseases. In contrast to the structured nature of, for instance, blood parameters, unstructured free text information is harder to analyze. Hence, research focuses on exploring NLP and LLMs for this task, which has been shown promising: improving patient outcomes [8], extracting valuable insights from clinical notes for transforming unstructured clinical notes into a structured form, identifying relevant clinical events to foster clinical decision systems [9]. Clinical NLP has also been leveraged to automate coding tasks and identify patient cohorts for research [10].

LLMs have shown significant promise in extracting and summarizing key information from unstructured clinical texts. Models such as FLAN-T5 and Mistral-Instruct have been tailored for medical applications by training on extensive datasets of medical literature and clinical notes [11]. These models can assist in information retrieval, summarization, and medical question answering. While promising, challenges remain in ensuring model generalizability, potentially hindering their utility for the envisioned use [11].

However, the application of NLP and LLMs in the broad area of dysphagia is largely underexplored. NLP methods have been utilized to analyze esophagogastroduodenoscopy (EGD) procedures, as outlined by recent work (e.g., [12]). The study indicated that employing NLP instead of administrative codes, e.g., ICD-10 codes, provides a more accurate identification of dysphagia as a common indication for EGD procedures. Segura et al. present an EHR-based study investigating the timing between symptom onset and disease-related outcomes in amyotrophic lateral sclerosis (ALS) [13]. Here, clinical NLP demonstrated potential in extracting and analyzing real-world evidence within the ALS population, where dysphagia is one of multiple symptoms. The study found that NLP could help reduce a delay between symptom onset and initial ALS notation, potentially enabling patients to receive earlier treatment.

In the broader sense, the analysis of EHRs from dysphagia patients with machine learning tools could identify dysphagia and aspiration pneumonia [14]. The authors showed that their ML models demonstrate strong discriminative performance, suggesting the potential for integration into routine clinical screening practices.

## III. Methodology

The study analyzes a comprehensive, fully anonymized, monocentric EHR dataset comprising only dysphagic pathological cases. Among other detailed information, the patient’s records of interest are stored in unstructured free text and cover diagnostic information as well as symptomatic reports of the patient. We want to analyze whether there are similarities between dysphagic patients stored in the EHR, i.e., by similar diagnoses, and summarize them on this basis.

### A. Dataset

Our EHR dataset consists of 486 dysphagic patients, who all reported dysphagic symptoms or were diagnosed with dysphagia and presented for instrumental swallowing assessment. The dataset contains information about the patient’s year of birth, age, gender, race, ethnicity, category of the disease primarily responsible for the patient’s dysphagia, medical diagnoses associated with the instrumental exam of the visit, medications, comorbidities, number of videofluoroscopy studies, and whether the person received a service from a speech-language pathologist (SLP) or not. The patients were all treated and recorded at the clinic Our Lady Of The Lake in Baton Rouge, USA. All data were acquired in accordance with IRBAM-21-0625. Additionally, the data was labeled by two experienced SLP experts, who agreed on one category for the patient’s source of dysphagia is neurogenic, neurodegenerative, cancer, and none of the aforementioned. Since a patient may have more than one diagnosis, we manually categorized the main cause of the patient’s presentation into one of the four categories. In this study, we only use the information about the associated medical diagnoses, which is written in unstructured free text. This implies that this information varies in length, level of detail, and information content. To verify the categorization ability of our NLP algorithms, we use the information about the manually labeled category from our medical experts as ground truth.

### B. Natural Language Processing

NLP incorporates techniques to understand human languages, which can range from simple counting techniques to semantic parsing [15]. Before applying NLP, we preprocessed the text to enhance uniformity within the unstructured free text form data by (i) removing special characters, (ii) removing stop words using the Natural Language Toolkit (NLTK) [16], and (iii) lowering all characters. Next, we converted the text representation to a mathematical one by encoding the text as a multidimensional vector. Via so-called embeddings, we aim to investigate semantic similarities between medical diagnoses. For the text-to-mathematical conversion, we utilize bidirectional encoder representations from transformer (BERT) [17] in its foundational version, alongside distinct fine-tuned variants including Med-BERT (MB) [18] trained on electronic health records, Clinical BERT (CB) [19] fine-tuned on clinical notes, and the Word2Vec (W2V) [20] approach. BERT uses the transformer architecture, which in particular employs several layers of self-attention mechanisms that allow the model to weigh the meaning of different words in relation to each other and in relation to their position in the text sequence [21]. Bidirectional means that the architecture uses both directions of the context to assess the word, i.e., before and after the word. This architecture and the bidirectional aspect allow BERT to dynamically understand the context of a word based on all other words and to capture speech patterns in different linguistic contexts. With these multidimensional embeddings, we approximate their representation with unsupervised manifold learning algorithms, specifically Uniform Manifold Approximation and Projection (UMAP) [22], allowing us to project this multidimensional information for each patient down to a single point in a two-dimensional coordinate system. To derive patient groups we apply a Density-Based Spatial Clustering of Applications with Noise (DBSCAN) [23] to identify and group patients with (presumably) similar dysphagic features. We measure the uniformity of the clustering ability mostly with the metric Intersection-over-Union (IoU). It calculates the number of elements of two groups, divided by the number of elements in both single groups. The IoU ranges from 0 to 1; the higher the value, the more both groups are alike.

### C. Large Language Models

LLMs demonstrate strong performance in NLP tasks due to their architecture, which enables them to capture long-distance relationships. Their training on large datasets establishes a comprehensive knowledge base that these models can effectively utilize [24]. The network architecture of these models is based on transformers, and the input is encoded in terms of a variety of language properties, e.g., the meaning of the word and the importance of the word in the sentence. Therefore, they work in a unidirectional way as causal language models, learning to predict the next word based on the preceding words. Through multiple iterations and layers of processing, NLP models utilize a self-attention mechanism that allows them to focus on different parts of the input. This enables the model to capture various linguistic properties, such as syntax and semantics, as previously mentioned [21]. These models then provide a mathematical understanding of the text as output, which can then be used for any NLP task [25]. Irrespective of misspellings and abbreviations, we aim to comprehend the primary information of the diagnoses and identify each patient’s most significant impairment by using the cognitive ability of LLMs. Therefore, we evaluate a variety of state-of-the-art LLMs, such as LLaMA 2 13B (Llama2) [26], PMC-LLaMA (PMC) [27], Mixtral $8\times 7$B (Mixtral) [28], and GPT-3.5 (GPT) [29]. First, we use these LLMs to analyze and interpret the data and generate a concise summary of the primary disease associated with the instrumental exam. The models are applied without additional hyperparameter tuning, and we use the same optimized prompt for all models to generate the output. Secondly, we use these models to categorize dysphagic pathologies. We utilize learned patterns and semantic similarities between wordings to distinguish between four previously defined categories, namely neurogenic, neurodegenerative, cancer, and other, see Fig. 3. Additionally, we investigate if the cognitive understanding of the LLM is sufficient to categorize dysphagic patients by adding prior knowledge to the prompt, e.g., Fig. 3 by including a definition for each category.

**FIGURE 1.**
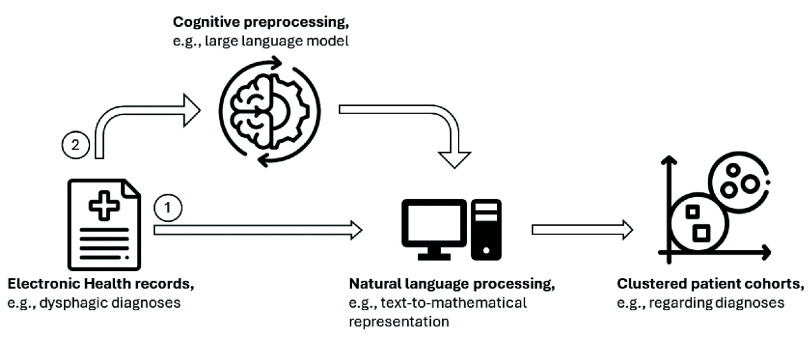
An overview of the process including two approaches of mining the text information of the patient diagnoses. The first approach only covers the preprocessing of the text data and uses the embeddings for clustering. In the second stream, we add an LLM and cluster based on the LLM-generated output.

**FIGURE 2.**
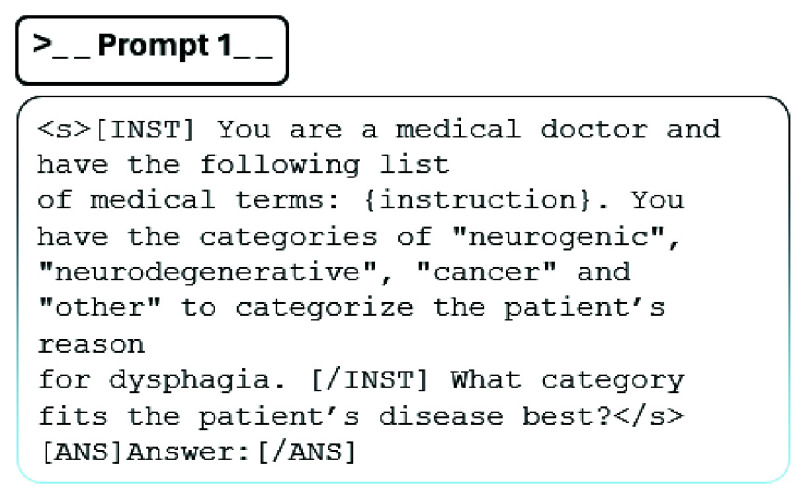
First prompt to categorize dysphagic patients in neurogenic, neurodegenerative, cancer, or none of the aforementioned categories.

**FIGURE 3.**
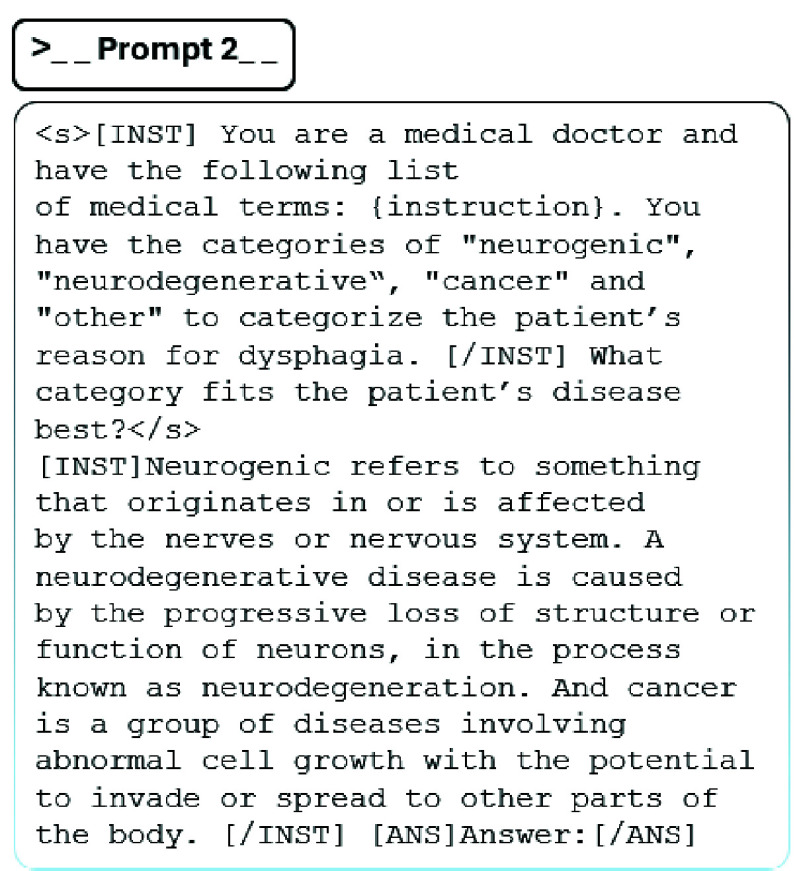
Second prompt to categorize dysphagic patients by including a definition for each category.

## IV. Results

### A. Analysis of Dataset Structure and Text Variability

**FIGURE 4.**
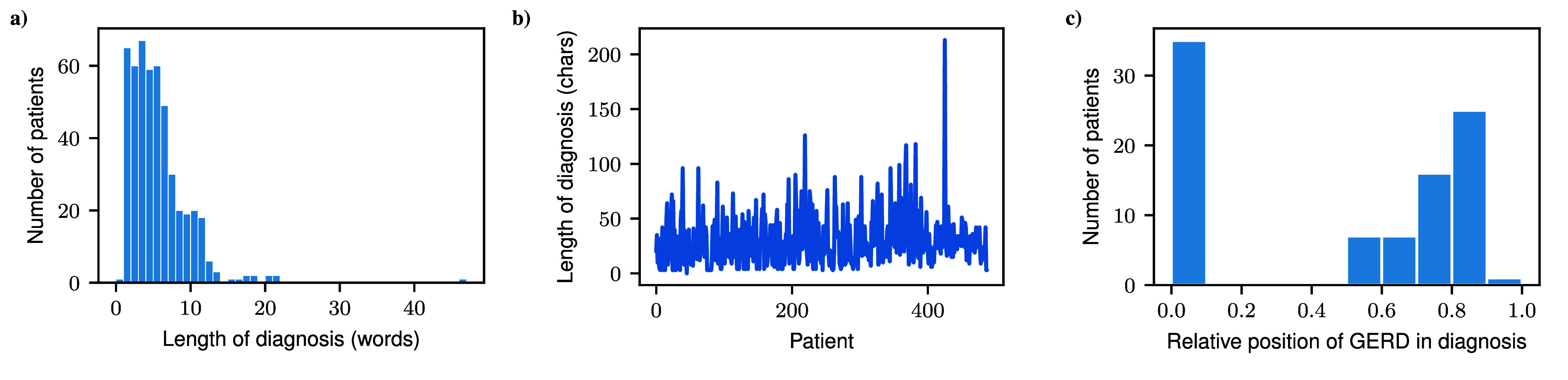
Analysis of our dataset, specifically of the associated medical diagnoses. a) represents the number of patients sharing the same number of words for the diagnoses. b) shows the variety of characters a diagnosis can contain. c) describes the number of patients, who share the symptom GERD in their diagnoses at any but also on the relative position in the text.

The dataset used in this study comprises records from 486 patients, each presenting a variety of pathological impairments. Since the diagnoses are presented as unstructured free text, there is high variability in their formulation. To familiarize ourselves with the data, we initially analyzed its structural properties. We analyzed these diagnoses by examining the total number of words and characters, as well as the structural format of the diagnostic text, as shown in Fig. 4. According to Fig. 4a), 91% of all diagnoses contain between 0 to 10 words, although a minor fraction may also include more than 40 words. Fig. 4b) illustrates the character count, revealing that some diagnoses exceed 200 characters. Additionally, as shown in Fig. 4c), the positioning of similar symptoms varies, with occurrences at both the beginning and end of the text. This variation in word order and length together with irregular positioning of diagnoses suggests inconsistencies in how information is recorded, which could potentially influence data interpretation. To address these issues, we apply NLP techniques to analyze the diagnoses and reduce biases related to text structure.

### B. Unsupervised Clustering Using Embeddings

**FIGURE 5.**
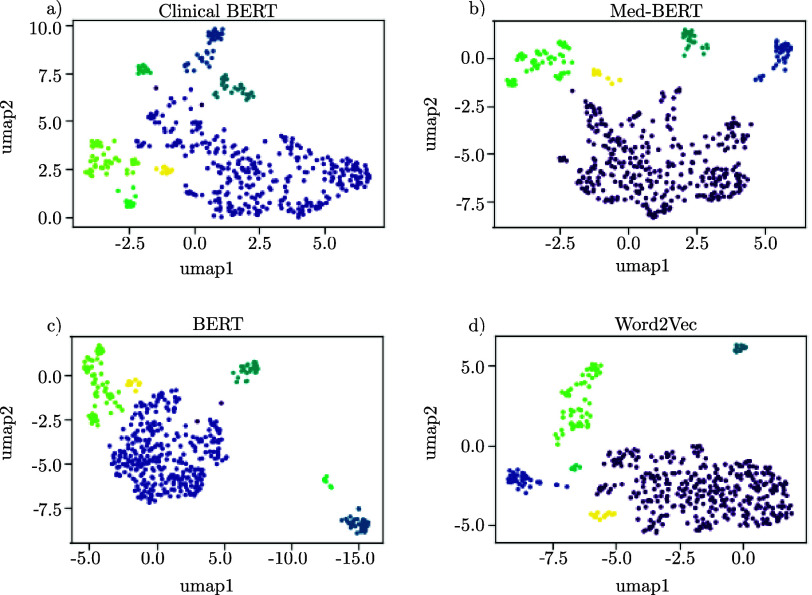
Analysis of the clustering ability of the patient’s diagnoses of four different embeddings after applying UMAP for dimensionality reduction and DBSCAN for class assignment. a) represents the text-to-vector conversion done with CB, b) with MB, c) with BERT, and d) with a W2V approach.

**FIGURE 6.**
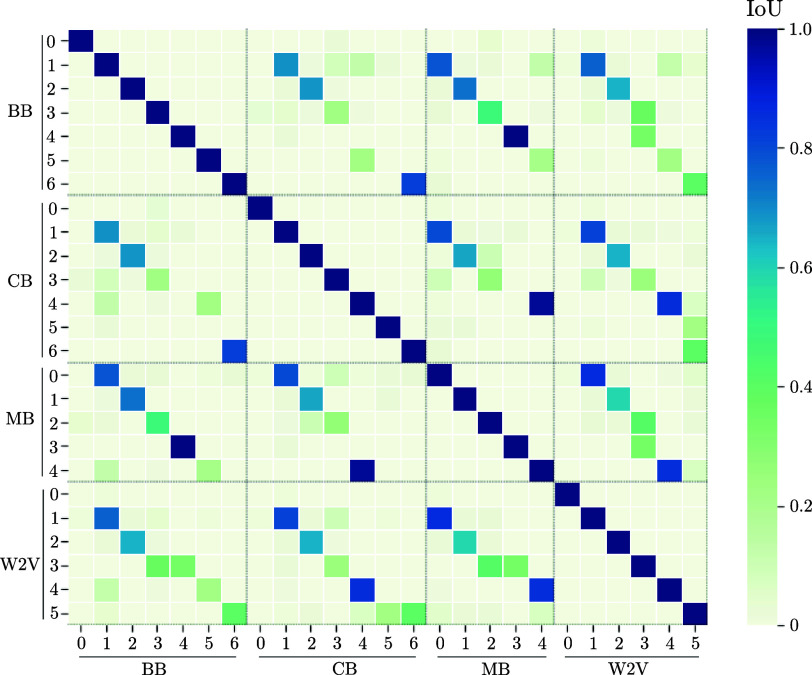
Correlation matrix of the IoU score of patients among the clusters of different embeddings. Each clustered group was used to calculate the IoU with all of the other embeddings.

To enhance our understanding of the recorded data, we employed various text embedding techniques, including three variants of BERT and one W2V model. These embeddings convert the preprocessed medical texts into mathematical vectors that represent the textual data, where one dot in Fig. 5 represents one patient’s diagnoses. We then applied UMAP to analyze these vectorized forms, as depicted in the referenced Fig. 5. Upon adjusting the UMAP’s hyperparameters, we observed that the clustered outputs for all four embeddings were dispersed and lacked clear differentiation among patient groups. Furthermore, a consistent number of clusters could not be established across the different embeddings. All embeddings show one large, main cluster with a lot of diffuse diagnoses and some smaller groups adjacent to the major core. To evaluate the extent of overlap among these clusters corresponding to different patient groupings, we computed the Intersection over Union (IoU) for the one-hot encoded vectors representing each cluster. The subsequent Fig. 6 illustrates the intersections among clusters from different embeddings. Notably, some clusters, such as the zeroth clusters of BERT, CB, and the W2V embeddings, showed no overlap with others. This lack of correlation could be attributed to these clusters containing only a small number of patients, which also minimizes opportunities for intersection with other groups. Overall, all other clusters of the various embeddings show high IoU values above 50 % with at least one cluster of all other embeddings. A further analysis of the patients in these clusters, unfortunately, shows no anomalies in the medical context. It can therefore be concluded that the embeddings cluster similar patient groups, but not that they are medically meaningful. In summary, our results suggest that these embeddings are not powerful enough to (i) encode efficiently patient information and (ii) generalize across the patient population.

### C. Impact of Cognitive Preprocessing on Clustering Efficiency

**FIGURE 7.**
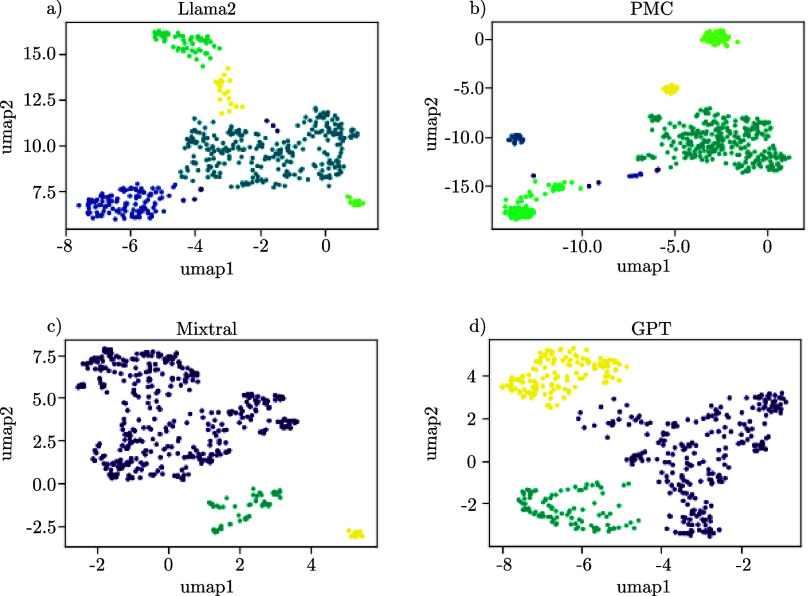
Analysis of diagnoses output generated with 4 different LLM models (Llama2, PMC, Mixtral and GPT). The cluster were build with UMAP after applying CB embedding.

**FIGURE 8.**
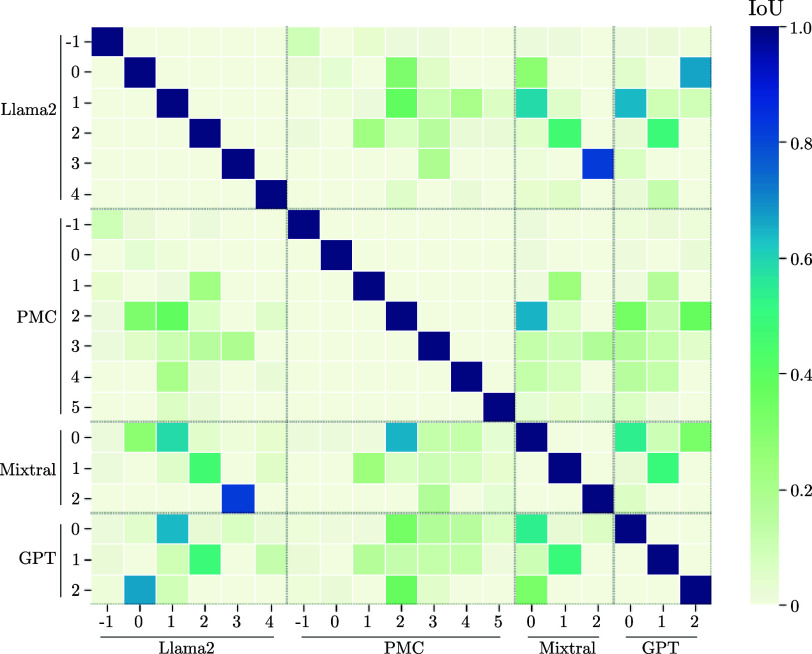
Correlation matrix of the IoU score of patients among the clusters of different LLMs. Each clustered group was used to calculate the IoU with all of the other clusters of the LLMs.

The text-based clustering approach showed limited correlation among the clusters, which is why we asked if we could enhance the output of the embeddings and leverage their full potential. To improve the distinguishability of different dysphagic pathologies through the embeddings, we incorporated a cognitive preprocessing stage to enhance the quality of the input data. Consequently, we used the patients’ primary associated diagnoses as input for four distinct LLMs. The LLMs present summarized versions of the patient’s symptoms and diagnoses by prioritizing primary impairments. Therefore, we utilized three open-source LLMs: Llama2, PMC, Mixtral, and one proprietary, continually refined LLM, namely GPT from OpenAI. Fig. 7 illustrates that clustering the LLM-generated diagnoses primarily results in a singular large patient group, except GPT, where the generated output yields two predominant clusters. Furthermore, we analyzed the IoU between all generated clusters, as shown in Fig. 8. We found IoU values above 0.5, especially for some clusters of GPT, Llama2, and Mixtral, while PMC showed hardly any overlap with clusters of other LLMs. The respective cluster sizes are detailed in Tab. 1, wherein diagnoses generated by PMC and Mixtral form a single cluster comprising over 300 patients. In Tab. 1, we computed the IoU between clusters generated by different LLMs. We identified the cluster from each LLM with the highest IoU with clusters from the other LLMs. Only a limited number of intersections exhibited notably high IoU scores. To discern cluster identities, we additionally analyzed the most frequent words within each cluster. For instance, the third cluster of Llama2 demonstrated an IoU score of 82 % with the second cluster of Mixtral, where the most frequent words mainly cover undefined diagnoses. For PMC, every cluster shares the symptom of gastroesophageal reflux disease, also known as GERD. GERD is a digestive disorder characterized by chronic acid reflux [30]. This observation suggests that PMC may not have effectively summarized the unstructured data to produce diagnoses that are conducive to unambiguous clustering. A better clustering of similar diagnoses can be shown by the second cluster of GPT and the zeroth cluster of Llama2. For these clusters, the IoU score is 66 % and the key values of both clusters also relate strongly to cancer-related diagnoses.

**TABLE 1:** Representation of LLM’s Cluster After Applying CB as Embedding, UMAP and DBSCAN. The Most Intersecting Cluster Group of the Other LLMs are Visualized by Highlighting the Cluster Number in Bold. The IoU is Given in Percentage for Each Cluster Combination

		Llama2	PMC	Mixtral	GPT	size	most frequent words in cluster
Llama2	-1		-	-	-	7	[’cancer’ ‘lung’ ’surgery’ ‘esophagitis’ ’bladder’]
	0		2 31%	0 28%	2 66%	114	[’scc’ ‘tongue’ ‘scca’ ‘carcinoma’ ’cell’]
	1		2 39%	0 59%	0 64%	270	[’dysphagia’ ‘gerd’ ‘acdf’ ‘throat’ ’foods’]
	2		1 22%	1 47%	1 49%	62	[’gerd’ ‘dysphagia’ ’hernia’ ‘htn’ ’tia’]
	3		3 19%	2 82%	-	16	[’nan’ ’cough’]
	4		-	-	1 12%	19	[’stricture’ ‘gerd’ ’esophageal’ ‘stenosis’ ’mvc’]
PMC	-1	-		-	-	5	[’lung’ ‘gerd’ ‘cancer’ ‘t1n2m0’ ’rbot’]
	0	-		-	-	6	[’cancer’ ‘tonsil’ ‘tongue’ ‘t4n2mx’ ’t3n2bmo’]
	1	2 22%		1 24%	1 17%	20	[’gerd’ ‘acdf’ ’esophageal’ ‘c3’ ’c4’]
	2	0 31%		0 65%	0 33%	301	[’scca’ ‘scc’ ‘gerd’ ‘tongue’ ’carcinoma’]
	3	1 11%		0 12%	0 18%	79	[’gerd’ ‘nan’ ‘scc’ ‘cancer’ ’cva’]
	4	1 20%		0 12%	0 15%	59	[’dysphagia’ ‘throat’ ‘foods’ ‘gerd’ ’food’]
	5	-		-	-	18	[’dementia’ ‘cva’ ‘gerd’ ‘lewy’ ’body’]
Mixtral	0	0 28%	2 65%		0 53%	410	[’dysphagia’ ‘scca’ ‘scc’ ‘gerd’ ’tongue’]
	1	2 47%	1 24%		1 50%	63	[’gerd’ ‘dysphagia’ ‘foods’ ‘throat’ ’esophageal’]
	2	3 82%	3 18%		-	15	[’nan’ ‘disorder’ ’c5’ ’acdf’]
GPT	0	1 64%	2 33%	0 53%		256	[’dysphagia’ ‘acdf’ ‘cancer’ ‘dementia’ ’nan’]
	1	2 49%	1 17%	1 50%		100	[’gerd’ ‘dysphagia’ ‘throat’ ‘foods’ ’stricture’]
	2	0 66%	2 37%	0 32%		132	[’scc’ ‘scca’ ‘tongue’ ‘cell’ ’carcinoma’]

**FIGURE 9.**
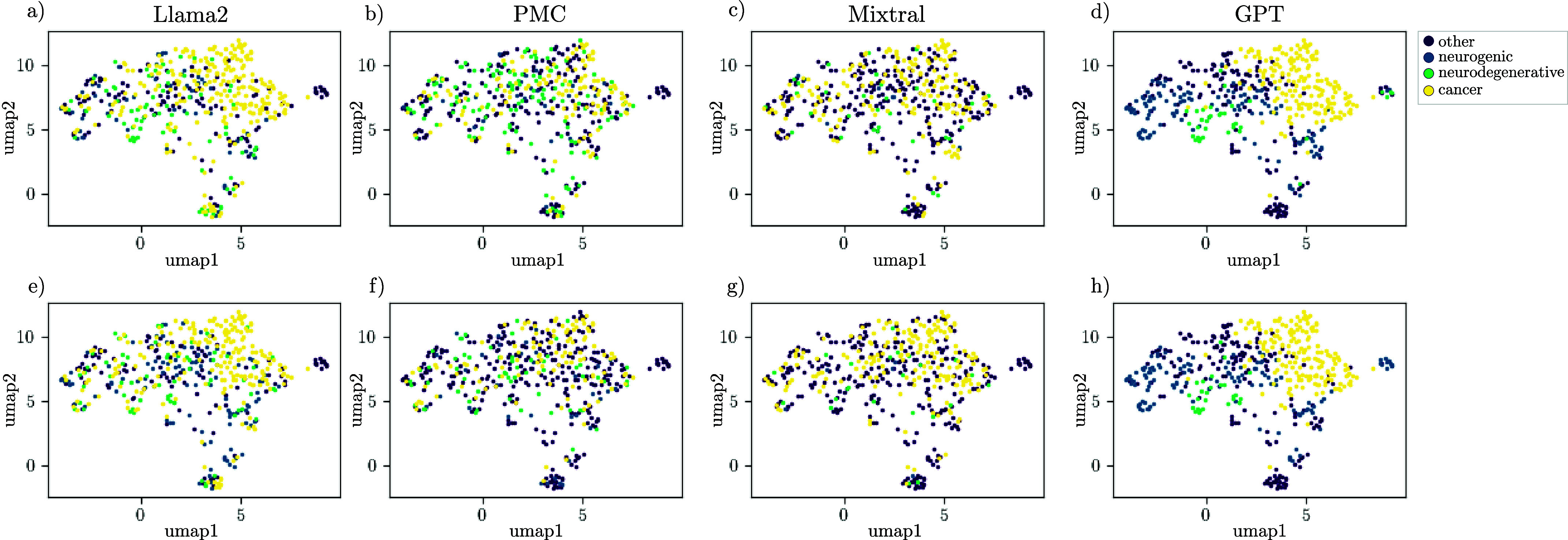
Analysis of the categories neurogenic, neurodegenerative, and all others assigned by the four different LLMs (Llama2, PMC, Mixtral, and GPT) based on the patient’s medical diagnoses. a) to d) present the performance of the respective LLMs to categorize without additional prior knowledge and fine-tuning of the models. e) to f) show the performance of the model including definitions for the single categories to support the category decision.

The evaluation of the preprocessing method, particularly the diagnoses summarization, proved some effect for the closed-source LLM but showed huge disparities among other LLMs. This observation suggests that the efficacy of clustering may be hindered by inconsistencies in the integration of dysphagic diagnoses across LLMs, as well as the diversity of diagnoses themselves. Consequently, we aimed to categorize patients based on their primary associated disease, particularly focusing on what likely caused dysphagia symptoms. Here, we delineated four primary categories: neurogenic, neurodegenerative, cancer, and other for patients not fitting into the aforementioned categories. A ground truth was generated by experts. First, we tasked our LLMs with generating a category following the first prompt, as detailed in Fig. 3. The prompt instructed the LLMs to categorize each diagnosis. Figures 9 a) to d) depict the patient diagnoses structured using MB embeddings, with categories indicated by colorization. In Fig. 9 a) to d), we observe that Llama2 and GPT exhibit promising results in clustering cancer-related diagnoses, while other categories appear dispersed. GPT demonstrates additional capability in discerning minor neurogenic subgroups. PMC and Mixtral encounter challenges in distinguishing between neurogenic and neurodegenerative diseases, promoting us to consolidate categories into neuro-related, cancer, and other. Figure S1 highlights the strong performance of Llama2 and GPT, particularly in distinguishing between cancer-related and non-cancer diagnoses. Conversely, PMC and Mixtral struggle to differentiate between neuro-related and other categories.

### D. Do LLMs Understand the Nuances of Dysphagia?

The limited ability of LLMs to differentiate between neurogenic and neurodegenerative diagnoses led us to investigate whether adding category definitions to the prompt, as shown in Fig. 3, would improve performance. Figures 9 e) to h) present the LLMs’ performance when additional information about the categories is included. Despite this modification, the results did not show improvement compared to those obtained without prior knowledge. Both Mixtral and PMC, which have been specifically fine-tuned on medical data, encountered difficulties in accurately classifying patient diagnoses within the provided categories, regardless of the presence or absence of additional category information.

**FIGURE 10.**
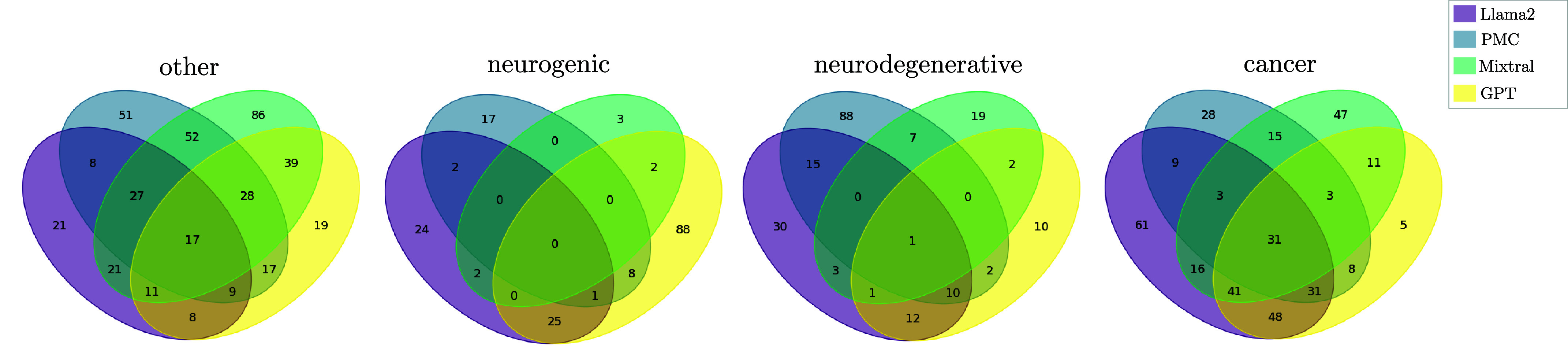
Analysis of the categorization ability of dysphagia diagnoses into other, neurogenic, neurodegenerative, and cancer of four different LLMs, e.g., Llama2, PMC, Mixtral and GPT.

**FIGURE 11.**
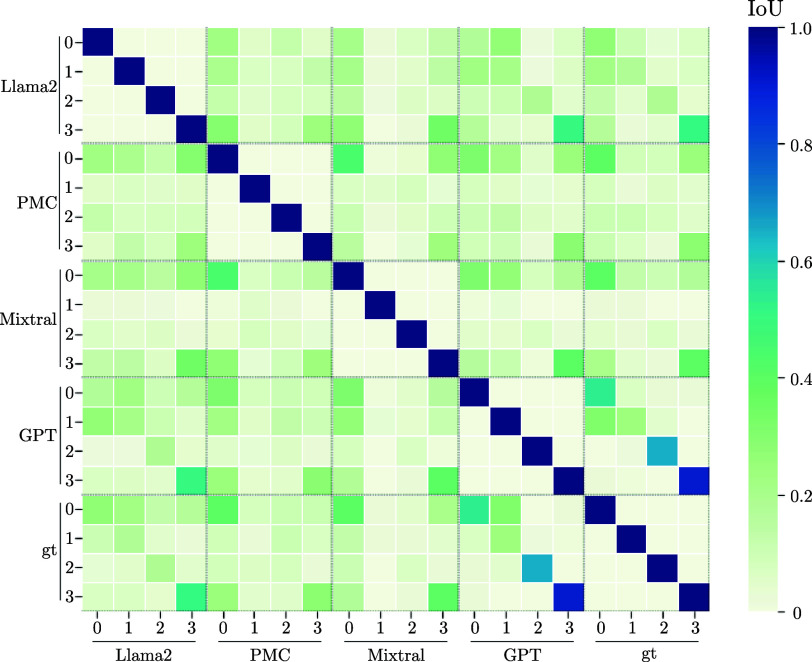
Analysis of the categorization ability of dysphagia diagnoses into other, neurogenic, neurodegenerative, and cancer against ground truth (gt, i.e., classification of our medical experts). The overlap of the LLMs and the ground truth is measured using the IoU.

Providing category definitions did not significantly enhance performance. Therefore, we aimed to quantify the number of similarly categorized patients to establish a primary categorization. In Fig. 10, comparisons among LLMs showed that in the *other* and *cancer* categories, 17 and 31 patients, respectively, were consistently categorized across the models. However, there was little overlap in the neurogenic and neurodegenerative categories. We compared the classifications from all LLMs with the ground truth established by our medical experts for dysphagia diagnoses. Initial analysis indicated that GPT performed well in categorization compared to open-source LLMs, even those trained specifically on medical data. Figure. 11 illustrates an IoU exceeding 50 % for the *other*, *neurodegenerative*, and *cancer* categories. This suggests that with continuous fine-tuning, maintained closed-source models tend to outperform open-source models, including those explicitly trained on medical data.

## V. Conclusion

In this study, we explored different approaches for mining EHR data, e.g., dysphagic diagnoses, initially using so-called embeddings alone and later with cognitive preprocessing through LLMs.

The data attributes were limited to the information provided, and so we did not include additional details about the patients’ underlying conditions in our study. Future studies could investigate specific attributes that improve patient differentiation and thus improve both within-class and between-class categorization.

We found that embeddings solely were not powerful enough to distinguish dysphagic patients into distinct and meaningful groups. LLMs were also not able to identify relationships between diagnoses to enhance patient information, which made diagnosis clustering challenging. To address this, we adopted a category-based clustering approach, where explicitly medical-trained LLMs could not compete with closed-source models.

LLMs are not yet mature enough to be applied lightly to medical data, as they are still challenged by the different terminology and abbreviations used in medical diagnoses. Our analysis showed that clustering methods by leveraging embeddings could be applied to free unstructured data, but it did not yield clear distinctions between different underlying pathologies. This may be due to the multiple diagnoses associated with dysphagia [30], the limited power of embeddings, and highly variable, unstructured texts.

To overcome the limitations of embeddings, we used LLMs (i) to preprocess diagnoses by filling in missing understanding and (ii) to assign categorizations. In both approaches, the LLMs showed different degrees of drifting focus in text generation [11]. This is particularly evident in Tab. S1, where we had the individual LLMs generate definitions of the three categories.

The answers vary greatly between the models and in some cases no longer have anything to do with the task. This limited performance is partly due to the fundamental complexity of the medical data but is also known to be a general limitation of LLMs. This phenomenon is commonly known as hallucination in the context of large language models (LLMs). It occurs when the model generates information that cannot be verified by reliable sources or that has no factual basis in the training data [31].

Following a disease-specific clustering by using NLP as well as more sophisticated language processing using LLMs showed weak performance. Category-driven decision-making approaches with LLMs rather showed promising results, particularly for the closed-source model GPT. This shows that LLMs can be used for mining EHR and downstream applications but under very limited circumstances due to hallucination.

Cui et al. show the integration of a collaborative approach in the disease prediction task of the LLM. This covers an agent approach, which consists of two parts: to learn from its mistakes and to adapt to the challenges of the medical data [32].

Shi et al. additionally propose the EHR-Agent, which enriches the learning and guiding approach of LLMs by including a long-term memory to learn from the past [33].

However, the most important aspect that needs to be improved when applying LLMs in a medical context is the limitation of the hallucination factor. One form of addressing this problem is shown in [34], where the analysis of multimodal EHR data is tackled by a Retrieval Augmented Generation (RAG) approach. This includes the encoding of long-contextual clinical notes and time series data, but also the extraction of teaser-relevant medical entities. Verification against professional medical knowledge helps to ensure consistency and eliminate hallucinations.

This and further future advancements in LLMs and their expanding capabilities present an opportunity to handle EHR data more efficiently. This will be crucial as medical big data, particularly EHRs, continues to grow, necessitating robust tools for data analysis and categorization. Such tools could enhance treatment strategies for dysphagic patient cohorts and other groups.

## Supplementary Materials

Supplementary Materials
