# Supplementary material for: Unstructured Electronic Health Records of Dysphagic Patients Analyzed by Large Language Models
Source: IEEE J Transl Eng Health Med. 2025 May 19;13:237–45. doi: 10.1109/JTEHM.2025.3571255 (PMC12310174; doi:10.1109/JTEHM.2025.3571255)
Supplement: Supplementary Materials [file supp1-3571255.pdf]

Unstructured electronic health records of dysphagic patients analyzed by large language models

Luisa Neubig[1], Deirdre Larsen[2], Melda Kunduk3, Andreas M. Kist[1]

SUPPLEMENTARY INFORMATION

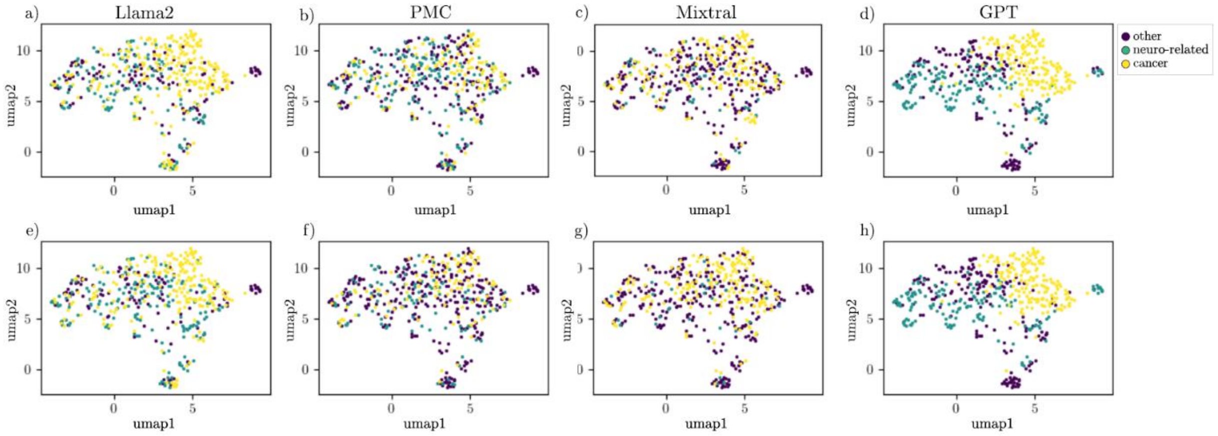

Fig. S1 Analysis of the categorization ability of four different LLMs,e.g., Llama2, PMC, Mixtral, and GPT by neglecting the detailed differentiation into neurodegenerative and neurogenic, but distinct between other, cancer and neurological. a)-d) present the performance of the respective LLMs to categorize without additional prior knowledge and finetuning of the models. e)-f) show the performance of the LLMs by including definitions for the single categories to support the category decision.

TABLE SI
GENERATED DEFINITIONS FOR EACH CATEGORY OF THE LLMS.

GPT-3.5	
neurogenic	pertaining to diseases caused by dysfunction of the nervous system
neurodegenerative	deterioration and loss of function of neurons in the brain or peripheral nervous systeme
cancer	a group of diseases characterized by the uncontrolled growth and spread of abnormal cells
LLaMA 2 13B	
neurogenic	Neurogenics refers broadly to diseases or conditions that affect nerve cells in either central (brain) , peripheral nervous system(spinal cord).
neurodegenerative	Neurodegenratve diseases referrto dysfunction or death of neurons in specific parts brain leading too cognitive decline and motor function impairment. Examples include Alzheimer's Parkinson , Huntington
cancer	A group(group)of diseases characterized by uncontrolled cell growth that spread throughout body
Mixtral 8x7B	
neurogenic	Involve progressive nervous system degeneration, including dementia (loss of cognitive function) or neuromuscular dysfunction affecting gait , movement coordination tremors weakness spasticity etc; examples include amyotrophic lateral sclerosis Alzheimer's Parkinson's Huntington chorea multiple-system atropy prion diseasessymptoms depend on type location severitv progressivenes

neurodegenerative	Neuro-muscular diseases of neuromusculoskeletal system, characterized by progressive loss or dysfunction in peripherical nerves. It includes Amyotrophic laterals sclerosis (ALA), Multiple Scleoris(MS)
cancer	Cancer refers specificallyto malignant neoplasms, or tumors. It can be caused by exposure of body tissues from chemicals such astobacco smoke; radiation in X-raysand gamma raysor even ultraviolet lightfrom sunlight etc.; viruses(suchas human papillomavirus), parasiteslike schistosomes that cause liver damage resultingin bladdercancer ; geneticmutationsthat occur spon
PMC-LLaMA	
neurogenic	The WHO classification of diseases (MCD) defines Neurological Disorder thusly. This section contains those conditions where there has been some form of acquired or congenital anomaly in either, structural development; chemical function transmission mechanisms; motor activities ; sensory responses; cognitive processes; mood and behavioural changes resulting from injury , tumours or premature ageings etc
neurodegenerative	Neuropathy, ALS, The amyotropic lateral sclerosisis also known as Lou Gehrig's Disease or Maladie de Charcot-Marie Tooth Type; it has no effective treatment at present but several trials of potential treatments are ongoing. It involves progressively weak muscles with sparing of eye movements
cancer	Carcinoma of head, face or neck : This group includes malignant neoplasms developing from epithelium lining surfaces in these regions. It excludes skin cancers (except eyelid) unless they involve underlying cartilage
